# Effect of Additional Light Curing on Color Stability and Degree of Conversion of Mono-Shade Resin Composites

**DOI:** 10.3390/polym18030403

**Published:** 2026-02-03

**Authors:** Fatih Bedir, Muhammet Karadaş, Makbule Gamze Atıcı Bedir, Alper Özdoğan

**Affiliations:** 1 Department of Restorative Dentistry, Faculty of Dentistry, Recep Tayyip Erdogan University, Rize 53020, Türkiye; muhammet.karadas@erdogan.edu.tr; 2 Rize Oral and Dental Health Center, Rize 53020, Türkiye; mgamzeatici@windowslive.com; 3 Department of Prosthodontics, Faculty of Dentistry, Atatürk University, Erzurum 25240, Türkiye; alper.ozdogan@atauni.edu.tr

**Keywords:** whiteness index, CIEDE 2000, ATR-FTIR, polymerization, light-curing unit

## Abstract

This study aims to examine the effect of additional light curing on the color stability and degree of conversion (DoC) of mono-shade resin composites cured using different light curing units and irradiation levels. Sixty-six disk-shaped samples were prepared for each of the mono-shade (Omnichroma/OC, Vittra APS Unique/VU) and multi-shade resin (Clearfil Majesty ES-2/CME) composites. The samples were randomly divided into three groups and cured for 20 s according to: (1) QTH at 800 mW/cm^2^ (16 J/cm^2^), (2) LED at 1000 mW/cm^2^ (20 J/cm^2^), and (3) 1400 mW/cm^2^ (28 J/cm^2^). After polishing, half of the samples in each group were exposed to additional light curing. Color parameters were measured at baseline and after 28 days of immersion in a coffee solution. CIEDE2000 color (∆E_00_) and Whiteness Index (ΔWI_D_) changes were used to assess color stability. ∆E_00_ and ΔWI_D_ were compared with the perceptibility and acceptability threshold. Mono-shade composites exhibited lower DoC with higher ΔE_00_ and ΔWI_D_ changes compared to the multi-shade composite. Mono-shade composites showed clinically unacceptable color and whiteness changes. Additional light curing performed using the same protocol both before and after polishing did not contribute to the color/whiteness stability and DoC of either mono-shade or multi-shade resin composites.

## 1. Introduction

Nowadays, increased esthetic expectations have significantly increased the use of tooth-colored resin composites [1]. To obtain an esthetically compatible resin composite restoration, it is often necessary to use multiple resin composites with different shade options to match the color of the natural tooth. The use of resin composites with the layering technique further complicates an already complex treatment process, prolongs the treatment time, and increases the overall cost [2]. To overcome these challenges, mono-shade dental resin composites containing nanofiller particles have been developed. Thanks to the chameleon effect, these materials provide better color matching with the surrounding tooth structure, eliminating the need to use multiple dental resin composites in different shades [3,4]. Thus, without the need to determine the shade of tooth in aesthetic restorations, a restoration compatible with the shade of the tooth can be made in a short time by using mono-shade dental resin composites [5].

Despite advancements, dental resin composites still have significant disadvantages such as polymerization shrinkage, bulk fracture, and discoloration [6]. Discoloration in dental resin composites can occur due to both extrinsic and intrinsic factors [7,8]. Inadequate curing, temperature changes, water absorption, adsorption of colorants in food and beverages, poor oral hygiene, and smoking are responsible for extrinsic discoloration [8], while resin composite properties such as filler particles, the resin matrix, and photoinitiators are responsible for intrinsic discoloration [7,8,9]. Since inadequate color stability can negatively affect treatment success and patient satisfaction, it can also reduce the lifespan of esthetic resin composite restorations and cause the dentist to decide to replace the restoration [10].

To activate the photoinitiator in dental resin composites and create a highly cross-linked structure, it is necessary to apply light with adequate energy intensity and the appropriate wavelength [11]. Insufficient curing can lead to an increased amount of residual monomers, resulting in compromised biocompatibility, increased water absorption, and the deterioration of mechanical and optical properties [12].

A study in the literature reported that additional light curing after polishing reduced discoloration in various resin composites (nanofill, nanohybrid, microhybrid, and bulk-fill) [13]. However, whether this is related to the degree of conversion (DoC) of the dental resin composite is not clearly explained by the authors. However, it is also curious whether the color stability of mono-shade composites, which have been reported to have lesser color stability than multi-shade composites in previous studies [5,14], will increase with additional light curing after polishing.

This study aimed to examine the effect of additional light curing after polishing on the color stability and DoC of mono-shade composite resins cured with light-emitting diode (LED) and quartz tungsten halogen (QTH) light units at different light irradiance levels. In this study, we tested the hypothesis that additional light curing after polishing would not significantly affect (1) the color stability and (2) the DoC of mono-shade composites.

## 2. Materials and Methods

### 2.1. Study Design

**Figure 1:**
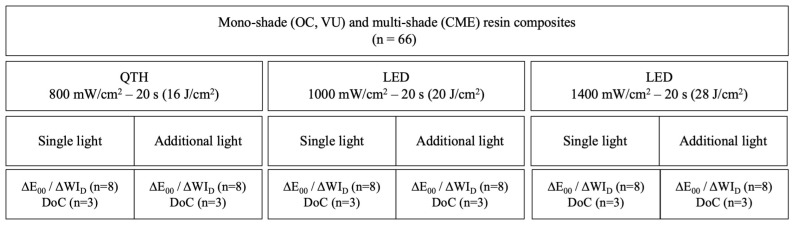
Study design (*n* = 198).

This study was designed with 18 groups based on the combination of three factors: (composite resins: three levels; light curing methods: three levels; additional light curing: two levels). The study groups are shown in Figure 1. The ∆E_00_ and WI_D_ indexes of the samples were measured using a spectrophotometer, and the DoC was assessed by attenuated total reflection–Fourier transform infrared spectroscopy (ATR-FTIR; Spectrum 100, PerkinElmer, Waltham, MA, USA).

### 2.2. Samples Preparation

A total of 198 disk-shaped samples (*n* = 66/per group; diameter = 6 mm, and thickness = 2 mm) were obtained from the three composite resins [Clearfil Majesty ES-2/CME (Kuraray Noritake, Tokyo, Japan), Omnichroma/OC (Tokuyama Dental, Tokyo, Japan), and Vittra APS Unique/VU (FGM, Joinville, SC, Brazil)]. Table 1 provides details on the composition of the materials used in this study. The teflon mold and transparent mylar strip were positioned onto a glass slide, and then composite resins were placed in a single layer in a teflon mold. The composite resin was placed in a teflon mold and compressed with another transparent mylar strip and a glass slide to prevent the formation of an oxygen inhibition layer and to obtain a smooth, clinically relevant surface. The samples were cured using two light curing units (QTH, BlueLuxer M-835, Monitex, Taiwan; LED, VALO Cordless, Ultradent, South Jordan, UT, USA) via three different methods (*n* = 66): (1) QTH at 800 mW/cm^2^ for 20 s (16 J/cm^2^), (2) LED at 1000 mW/cm^2^ for 20 s (20 J/cm^2^), and (3) 1400 mW/cm^2^ for 20 s (28 J/cm^2^). The light output power of the light curing units was checked using a radiometer (Hilux, Benlioğlu Dental Inc., Ankara, Turkey).

The finishing and polishing process utilized a two-step system containing diamond particles (Clearfil Twist Dia, Kuraray Noritake Dental Inc., Okayama, Japan). The finishing and polishing of the composite resins were performed under water cooling at 10,000 rpm for 20 s to prevent microcracks [5]. The samples were then randomly divided into two groups. Half of the samples were re-cured with a light-curing unit, while the other half were not. Additional light curing was applied for the same duration with the same light-curing unit as at the beginning. After that, the samples were cleaned in an ultrasonic cleaner for 5 min and then kept in 37 °C distilled water for 24 h [15]. The preparation of the samples, the finishing/polishing processes, and the additional light curing were performed by a single researcher and always on the same side of the samples.

### 2.3. Color Differences and Whiteness Index

Baseline color measurements for each sample in the group (*n* = 8) were obtained using a spectrophotometer (VITA Easyshade Advance, Zahnfabrik, Bad Säckingen, Germany). The spectrophotometer was calibrated before each measurement. For color assessment, the probe was positioned perpendicular to the center of each sample, and measurements were made on a gray background (Munsell N7 neutral gray color) [16,17]. The color of each sample was measured three times, and the average color coordinates of the three measurements were recorded [16,17]. A single researcher carried out the measurements using standardized D65 light illumination (Judge QC, X-Rite, Grand Rapids, MI, USA). Following the baseline color measurement, the samples were immersed in a coffee solution (Nescafe Classic Single Bag, Nestlé, Girona, Spain) at 37 °C for 28 days [18]. Freshly prepared coffee solution was used daily to prevent bacterial or yeast contamination. The coffee solution was obtained by dissolving 2 g of coffee powder in 200 mL of boiled water without adding sugar or milk, according to the manufacturer’s instructions. Before the samples were immersed in the coffee solution, the solution temperature was 37 °C. The samples were immersed in the coffee solution for 28 days, then washed with water and air dried. The color of each sample was then measured again using a spectrophotometer, as described above. The color difference was calculated according to the CIEDE2000 (∆E_00_) formula provided below [19,20]:

$${\Delta E}_{00}^{{}^{\circ}}=\sqrt{{\left(\frac{{\Delta L}^{\prime }}{K_{L}S_{L}}\right)}^{2}+{\left(\frac{{\Delta C}^{\prime }}{K_{C}S_{C}}\right)}^{2}+{\left(\frac{{\Delta H}^{\prime }}{K_{H}S_{H}}\right)}^{2}+R_{T}\left(\frac{{\Delta C}^{\prime }}{K_{C}S_{C}}\;\right)\left(\frac{{\Delta H}^{\prime }}{K_{H}S_{H}}\right)}$$

where ∆L′ is the difference in lightness, ∆C′ is the difference in chroma, and ∆H′ is the difference in hue. S_L_, S_C_, and S_H_ are weighting functions to adjust the total color difference for variation in the location of the color difference pair in *L*′, *a*′, and *b*′ coordinates. The parametric factors K_L_, K_C_, and K_H_, are correction terms for experimental conditions. And, finally, R_T_ is a rotation function that accounts for the interaction between chroma and hue differences in the blue region [19,20]. K_L_, K_C,_ and K_H_ were set as 1.0 for this study. The color difference was analyzed against 50:50% perceptibility and acceptability thresholds (PT = 0.81 ∆E_00_ units and AT = 1.77 ∆E_00_ units) [21,22].

The whiteness index for dentistry (WI_D_), which is based on CIELAB and has a linear formulation, was obtained according to the following formula [23]:

$${WI}_{D}={0.55L}^{*}-{2.32a}^{*}-{1.100b}^{*}$$

Lower WI_D_ values, including negative values, refer to darker samples and higher WI_D_ values to whiter samples. Differences in WI_D_ (ΔWI_D_) were obtained by calculating the difference between the initial and final measurements [24]. ΔWI_D_ was analyzed against 50:50% perceptibility and acceptability thresholds (WPT = 0.61 ∆WI_D_ units and WAT = 2.90 ∆WI_D_ units) [23]. In line with previous studies, the perceptibility (WPT) and acceptability (WAT) threshold values were applied to the absolute values of ΔWI_D_ (|ΔWI_D_|) [25].

### 2.4. Degree of Conversion

The DoC of the samples (*n* = 3) was performed using Fourier transform infrared spectroscopy (FTIR; Spectrum 100, PerkinElmer, Waltham, MA, USA) with an attenuated total reflection (ATR) accessory with diamond crystal. The samples were placed on an ATR crystal holder to completely cover the crystal surface. All spectra were collected over the range of 650 to 4000 cm^−1^, with 32 scans and a spectral resolution of 4 cm^−1^. The ATR-FTIR data were analyzed using OriginPro 2021 software (OriginLab Corp., Northampton, MA, USA).

After standard baseline correction, the DoC was calculated by determining the ratio of the peak height absorbance intensity of aliphatic C=C at 1638 cm^−1^ to that of aromatic C=C at 1608 cm^−1^ in both uncured and cured resin composite samples, using the formula below [26]:

$$\mathrm{DoC}(\%)=\left[1-\frac{{\left({1638\;\mathrm{cm}}^{-1}/{1608\;\mathrm{cm}}^{-1}\right)}_{\mathrm{peak}\mathrm{heights}\mathrm{cured}}}{{\left({1638\;\mathrm{cm}}^{-1}/{1608\;\mathrm{cm}}^{-1}\right)}_{\mathrm{peak}\mathrm{heights}\mathrm{uncured}}}\right]\times 100$$

However, for some resin composites that do not contain Bis-GMA (OC and VU), the carbonyl (C=O) group with a peak at 1716 cm^−1^ was used as the internal standard due to the absence of a benzene ring structure, and the DoC was calculated using the following formula [27]:

$$\mathrm{DoC}(\%)=\left[1-\frac{{\left({1638\;\mathrm{cm}}^{-1}/{1716\;\mathrm{cm}}^{-1}\right)}_{\mathrm{peak}\mathrm{heights}\mathrm{cured}}}{{\left({1638\;\mathrm{cm}}^{-1}/{1716\;\mathrm{cm}}^{-1}\right)}_{\mathrm{peak}\mathrm{heights}\mathrm{uncured}}}\right]\times 100$$

### 2.5. Statistical Analysis

The most recent guidance on color measurements issued by the International Organization for Standardization (ISO/TR 28642:2016) [22] evaluated color differences using comparisons with 50:50% thresholds. Data from ∆E_00_, ∆WI_D_, and DoC were statistically analyzed using the three-way ANOVA (composite resin, light curing method, and additional light curing), and multiple comparisons were calculated by Tukey’s post hoc test. The data were analyzed with SPSS software (v29.0, IBM., Chicago, IL, USA). The statistical significance level was accepted as 0.05.

## 3. Results

### 3.1. Color Differences (ΔE_00_) and Whiteness Index (ΔWI_D_)

Table 2 and Table 3 present the means and standard deviations of color (∆E_00_) and whiteness change (∆WI_D_) for the resin composites. A three-way ANOVA revealed that the resin composite (*p* = 0.000 and *p* = 0.000, respectively) and light curing method (*p* = 0.027 and *p* = 0.000, respectively) significantly affected ΔE_00_ and ∆WI_D_, while the additional light curing (*p* = 0.066 and *p* = 0.209, respectively) did not significantly affect ΔE_00_ and ∆WI_D_. Double and triple interactions between factors also did not statistically affect ΔE_00_ and ∆WI_D_ significantly (*p* > 0.05).

Regardless of the additional light curing, in all light curing methods, mono-shade resin composites (OC and VU) showed statistically significant greater color (∆E_00_) and whiteness (∆WI_D_) changes compared to multi-shade composite resin (CME) (*p* = 0.000). Both mono-shade (OC and VU) and multi-shade (CME) resin composites exhibited significantly more extensive color and whiteness changes when cured with a QTH light-curing unit at 800 mW/cm^2^ for 20 s, regardless of additional light curing (*p* < 0.05).

**Figure 2:**
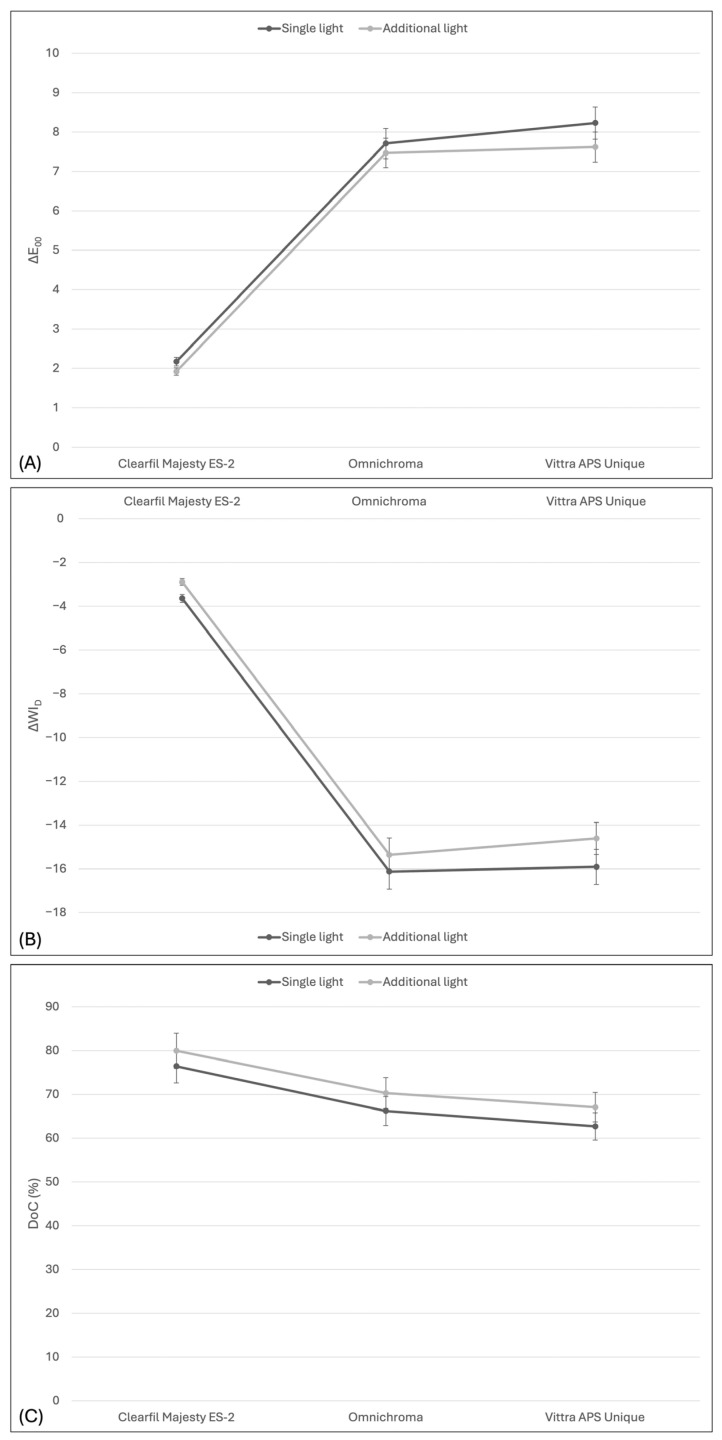
Line graph showing the mean ∆E_00_ (**A**), ΔWI_D_ (**B**), and DoC (**C**) of composite resins, regardless of light polymerization methods.

Regardless of the light curing method, mono-shade composites (OC and VU) showed significantly greater color (∆E_00_) and whiteness (∆WI_D_) changes compared to the multi-shade composite (CME) under additional light curing (*p* = 0.000). Statistically significant changes in ∆E_00_ and ∆WI_D_ were observed among single light-cured resin composites (*p* < 0.05). CME showed the lowest color (∆E_00_) and whiteness (∆WI_D_) change, while VU showed the highest change (Figure 2).

**Figure 3:**
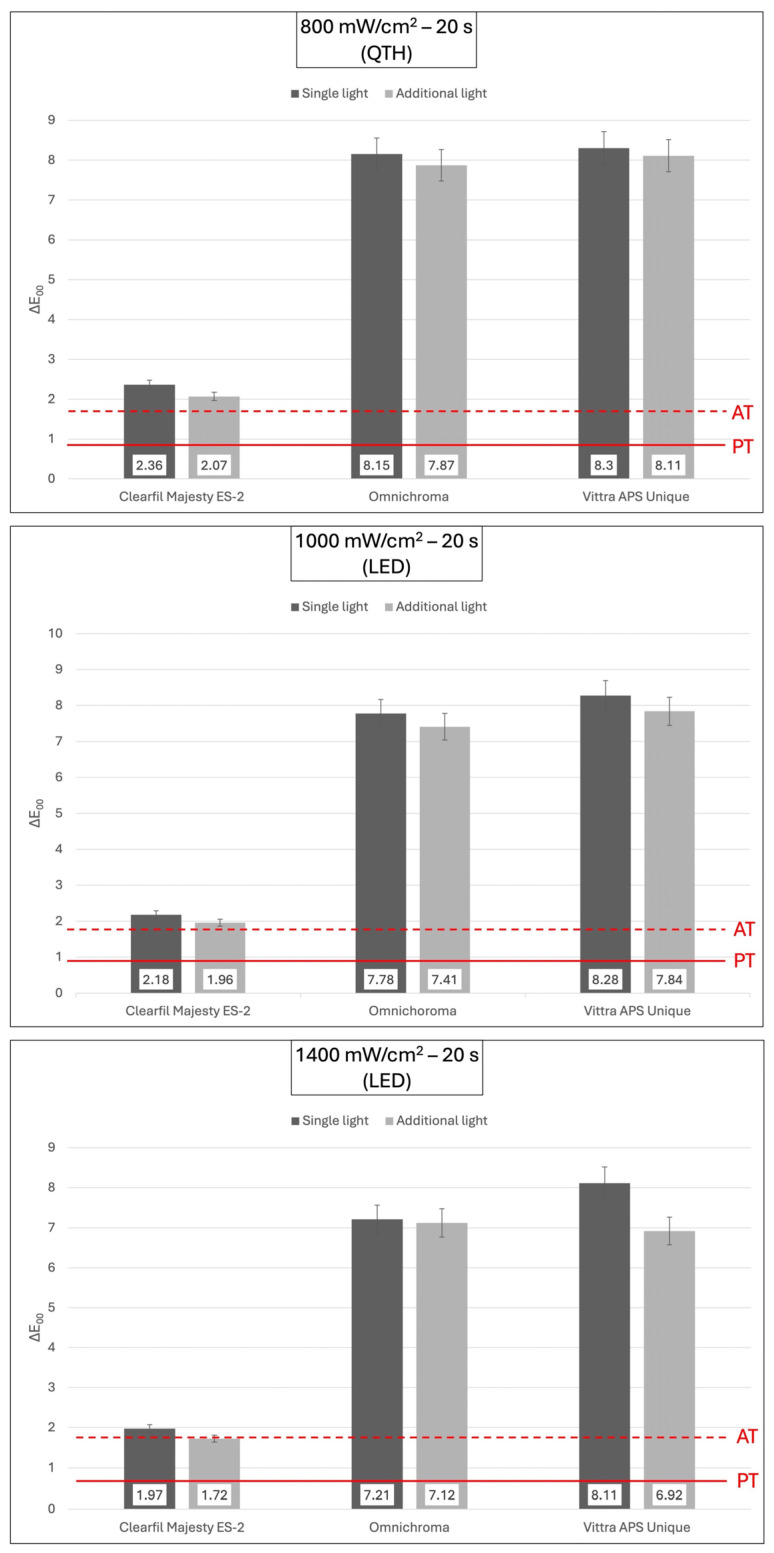
Mean ∆E_00_ values and standard deviations (SD) of composite resins polymerized in different light polymerization methods. The continuous line at 0.81 ∆E_00_ units represents the perceptibility threshold (PT) and the dashed line at 1.77 ∆E_00_ units represents the acceptability threshold (AT).

**Figure 4:**
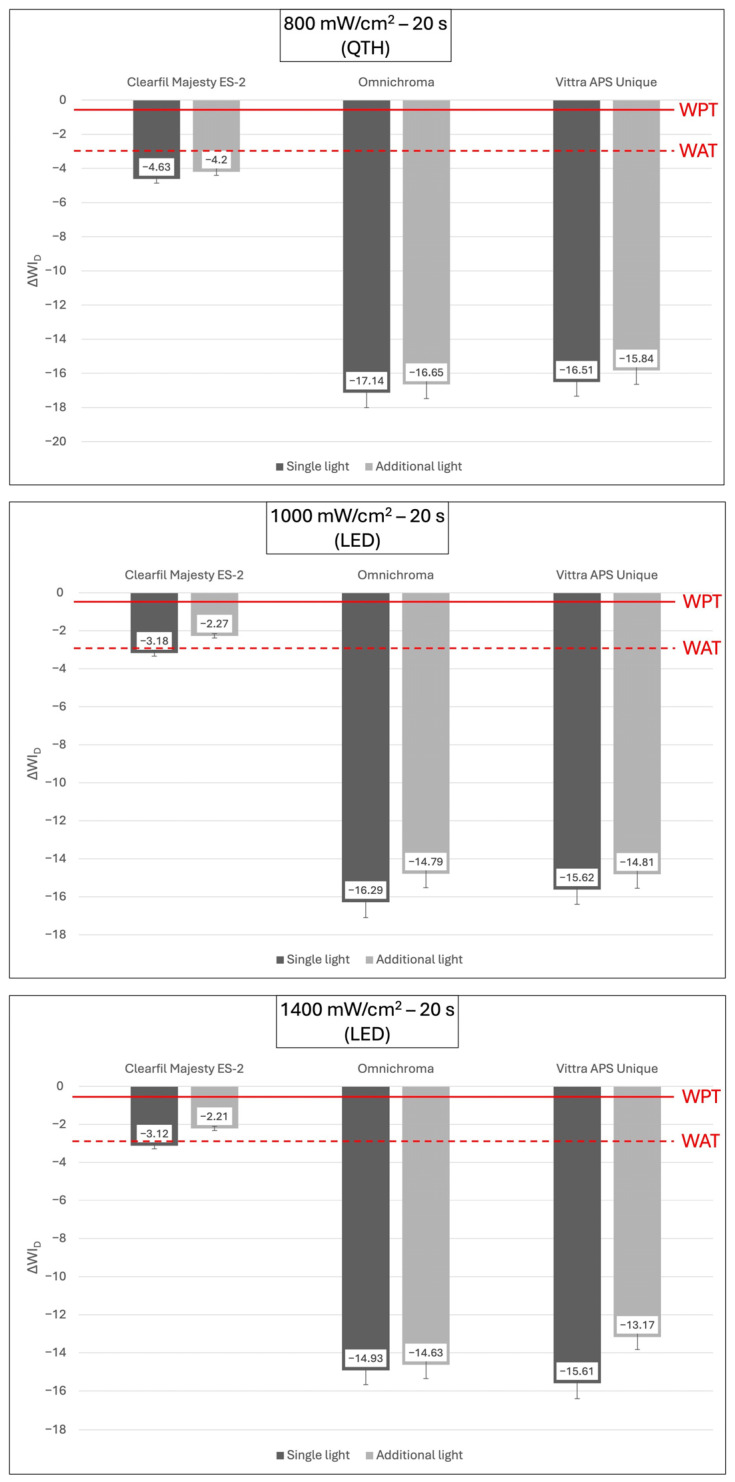
Mean ΔWI_D_ values and standard deviations (SD) of composite resins polymerized in different light polymerization methods. The continuous line at 0.61 ΔWI_D_ units represents the whiteness perceptibility threshold (WPT) and the dashed line at 2.9 ΔWI_D_ units represents the whiteness acceptability threshold (WAT). Threshold comparisons were performed using absolute ΔWI_D_ values. Negative ΔWI_D_ values indicate that specimens showed lower WI_D_ values at a later evaluation.

All the experimental groups showed a clinically perceptible color change above the PT value. Furthermore, all groups, except for the CME group, cured with additional light at 1400 mW/cm^2^ for 20 s exhibited clinically unacceptable color changes above the AT (Figure 3). Similarly, all groups showed perceptible whiteness changes above the WPT. However, only the CME with additional light curing at 1000 mW/cm^2^ and 1400 mW/cm^2^ for 20 s exhibited clinically acceptable whiteness changes below the WAT (Figure 4).

### 3.2. Degree of Conversion (DoC)

**Figure 5:**
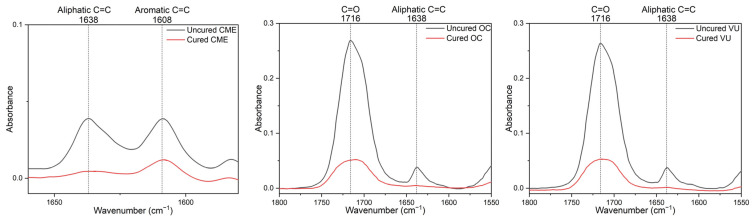
ATR-FTIR spectra for cured and uncured resin composites.

The representative ATR-FTIR spectra of both multi-shade and mono-shade resin composites before and after polymerization are shown in Figure 5.

Table 4 presents the means and standard deviations of DoC (%) for resin composites. A three-way ANOVA revealed that the resin composite (*p* = 0.001) significantly affected the DoC, while the light curing method (*p* = 0.661) and additional light curing (*p* = 0.209) did not significantly affect the DoC. The double and triple interactions between the factors were not statistically significant (*p* > 0.05) except for the double interaction between resin composite and light curing method (*p* = 0.014).

Regardless of additional light curing, VU cured at 800 mW/cm^2^ for 20 s exhibited the lowest DoC, while CME cured at 1400 mW/cm^2^ for 20 s showed the highest value. Regardless of the light curing method, both in the single light curing and additional light curing, mono-shade composites (OC and VU) were found to exhibit a significantly lower DoC compared to the multi-shade composite (CME) (Figure 2). However, no statistically significant difference was found between mono-shade composites (OC and VU).

## 4. Discussion

Recent advancements in dental restorative materials have led to the introduction of mono-shade resin composites, which simplify the layering technique and reduce the need for tooth color selection [5]. Both mono- and multi-shade resin composites may lose their initial color and whiteness over time [28]. High color stability in dental resin composites is important for the longevity of dental restorations [29]. Especially, anterior esthetic restorations with lesser color stability create aesthetic dissatisfaction in patients and negatively affect long-term treatment success [30,31]. Moreover, this condition is also considered by clinicians as an indication of the material’s aesthetic inadequacy [5]. For these reasons, investigating the color stability and the DoC of mono-shade resin composites cured using different light-curing units and irradiance levels, and subjected to the same additional curing protocol after polishing, will contribute to a better understanding of their clinical performance [32].

In this study, two null hypotheses were tested. The first was that additional light curing would not affect the color stability of mono-shade composites; the second was that additional light curing would not affect the DoC of mono-shade composites. According to the results of this study, the application of additional light curing did not affect both the color and whiteness stability, and the DoC of mono-shade composites. These results led to the acceptance of both null hypotheses.

In this study, mono-shade resin composites (OC and VU) showed greater color and whiteness changes than the multi-shade composite (CME), regardless of the light curing method and additional light curing. Consistent with this study, many studies in the literature have shown that mono-shade resin composites show more discoloration than multi-shade resin composites when exposed to various discolorants [5,14]. This finding is concerning for mono-shade resin composites, which are specifically developed to achieve rapid and effective color matching with the tooth being restored. The reason for the lesser color stability of mono-shade composites may be due to factors affecting color stability such as the chemical structure of the monomers in the resin matrices, the concentrations of monomers, photoinitiators, activators, and inhibitors, and the size and volume of inorganic fillers [7,8,9,29].

Composites with more hydrophilic resin matrices, due to their higher water sorption capacity, exhibit lesser color stability [33,34]. Resin matrices with Bis-GMA as the main monomer shows lower water absorption than resin matrices containing TEG-DMA, but higher water absorption than resin matrices containing UDMA and Bis-EMA [4]. Therefore, the type and proportion of monomers in the resin matrix has a direct impact on the color stability of resin composites as they affect water absorption [35]. Especially, the small and hydrophilic molecular structure of TEG-DMA exhibits higher mobility in water environments [36,37]. The greater color and whiteness changes observed in mono-shade resin composites in this study can be explained by the presence of the TEG-DMA monomer. Although the manufacturer states that VU is composed of a mixture of methacrylate monomers, most studies indicate that VU contains TEG-DMA and UDMA, similarly to OC [5,38]. Supporting this, no difference was found between mono-shade composites in terms of color and whiteness stability in this study. Additionally, only the multi-shade resin composite (CME), which showed greater color and whiteness stability in this study, contains Bis-GMA. The presence of Bis-GMA enhances the cross-link density of the polymer network [39,40], thereby improving both the mechanical properties and the color stability of the resin composite [41]. However, most manufacturers do not fully disclose the composition of resin composites. Therefore, it is very difficult to precisely identify the component responsible for the differences among resin composites [42].

As the amount of inorganic filler increases, the amount of the resin matrix decreases and therefore water absorption decreases [24]. Therefore, higher inorganic filler content in resin composites is associated with greater color stability [16]. However, in the present study, mono-shade resin composites (OC and VU), which have a higher inorganic filler volume than the multi-shade resin composite (CME), exhibited lesser color stability. The authors may explain this finding by suggesting that water absorption can still occur in composites with high inorganic filler content and low resin matrix volume. Therefore, hydrolytic degradation at the resin matrix and inorganic filler interface and, consequently, discoloration of the resin composite may occur [24,43,44].

It has been reported that total radiant exposure (J/cm^2^) per unit area is more important than light irradiance (mW/cm^2^) for the adequate curing of dental resin composites [45]. In the literature, there are different studies reporting the radiant exposure value required for the ideal curing of resin composites with a thickness of 2 mm as 12–24 J/cm^2^ [46], 10–11 J/cm^2^ [47,48], 16 J/cm^2^ [49], and 21–24 J/cm^2^ [50]. In this study, mono- and multi-shade resin composites were exposed to radiant exposure of 16 J/cm^2^ with a QTH light unit, and to 20 J/cm^2^ and 28 J/cm^2^ with LED light unit. As a result, a greater color and whiteness change was observed in the light curing method at 800 mW/cm^2^ for 20 s (16 J/cm^2^) regardless of resin composite and additional light curing. However, the light curing method did not affect the DoC of resin composites. Contrary to the present study, most studies that have investigated or not investigated the DoC have reported a close correlation between a higher DoC and greater color stability [13,41]. However, contrary to the current literature, the authors believe that the DoC of composites is not the only and/or the most important factor affecting color change. Lower radiant exposure may be sufficient to initiate cross-linking between monomers, yet it can also result in a less cross-linked, more linear polymer structure [51,52]. Such a polymer network is more susceptible to water sorption and pigment uptake, which may explain the increased discoloration observed despite acceptable DoC values [51,52].

The lack of improvement in the DoC following additional light curing may be explained by polymer network saturation and vitrification phenomena [53]. Once the initial curing phase provides sufficient radiant exposure to initiate extensive cross-linking, further monomer mobility becomes severely restricted due to the rapid increase in viscosity and formation of a rigid polymer network [54,55]. This vitrified state limits the diffusion of unreacted radicals and residual monomers, thereby preventing further DoC improvements, despite additional energy input [54,55]. Moreover, finishing and polishing procedures are known to effectively remove the superficial oxygen-inhibited layer, which is typically characterized by a lower degree of polymerization [56]. Therefore, additional light curing applied after polishing may have limited capacity to enhance the DoC, as the remaining polymer matrix is already highly cross-linked and diffusion controlled. These results suggest that once a critical radiant exposure threshold is achieved during initial curing, additional light curing using the same protocol does not result in measurable increases in the DoC of the resin composites evaluated in this study.

According to the manufacturer, the advanced polymerization system (APS) in VU is a combination of multiple photoinitiators, including camphorquinone [57]. Therefore, VU containing APS is expected to be more photosensitive than CME containing only camphorquinone. In contrast, VU demonstrated a lower DoC compared to CME. The manufacturer did not provide information on the photoinitiator content of OC. However, no significant difference was found between the DoC of the two mono-shade composites in this study. This finding suggests that polymerization efficiency is not governed solely by the complexity or number of photoinitiators, but rather by a combination of factors related to initiator chemistry, resin matrix composition, and light–material interactions. One possible explanation is a spectral mismatch between the emission profile of the light-curing units used and the absorption characteristics of the individual initiators within the APS. While multi-photoinitiator formulations are designed to spectral sensitivity, the specific absorption maxima and relative concentrations of these initiators are not fully disclosed by manufacturers, which may limit their effective activation under certain curing conditions [58]. In addition, increased initiator complexity may influence radical generation kinetics and polymer network development [59]. Excessive or heterogeneous radical production can promote early vitrification and restrict further monomer mobility, thereby limiting the extent of conversion despite adequate radiant exposure [59]. Furthermore, differences in the filler composition, translucency, and refractive index mismatch may affect light transmission within the composite, leading to greater light attenuation and reduced polymerization efficiency [60]. By contrast, the presence of Bis-GMA in CME may have contributed to higher cross-link density and more efficient polymer network formation [39]. Generally, these findings indicate that resin matrix composition and network-forming characteristics may play a more dominant role in determining final degree of conversion than photoinitiator system’s complexity alone.

The results of this study are, clinically, highly significant. In this study, mono-shade composites exhibited lesser color and whiteness stability as well as a lower DoC compared to multi-shade composites. In addition, the color and whiteness changes seen in mono-shade composites were clinically unacceptable. Furthermore, their tendency to become severely discolored over time suggests that these materials may require additional treatment in order to maintain their esthetic function. To overcome this issue, the additional light curing after polishing, which is an extra clinical procedure, showed no significant positive effect. Therefore, clinicians who wish to perform long-lasting anterior aesthetic restorations should consider the lesser color and whiteness stability of mono-shade composites and seek alternative methods of additional light curing after polishing in order to overcome this problem. In addition, in the present study, the same light curing protocol was applied both before and after the polishing procedure. It should be noted that the use of different additional light curing protocols in future studies may lead to different outcomes. Moreover, based on the results of this study, the high discoloration susceptibility of mono-shade composites appears to be primarily a material-related problem. Therefore, further research should be conducted to develop mono-shade resin composites that enable dental restorations to be performed without the need for color selection.

## 5. Conclusions

Within the limitations of this in vitro study, additional light curing performed using the same protocol both before and after polishing did not affect the color/whiteness stability and the DoC of both mono-shade and multi-shade resin composites. Mono-shade composites demonstrated lesser color and whiteness stability, as well as a lower DoC, compared to the multi-shade composite. In addition, the color and whiteness changes seen in mono-shade composites were clinically unacceptable. While the light curing method does not affect the DoC of resin composites, curing with a QTH unit at 800 mW/cm^2^ for 20 s has resulted in lesser color and whiteness stability in both mono- and multi-shade composites.

## Figures and Tables

**Table 1:** Composite materials used in this study.

Materials	Shade	Matrix	Filler	Filler Content wt%/vol%	Lot Number
Clearfil Majesty ES-2 (Kuraray Noritake, Tokyo, Japan)	A2	Bis-GMA, hydrophobic aromatic dimethacrylate, hydrophobic aliphatic dimethacrylate, and dl-camphorquinone.	Silanated barium glass filler, pre-polymerized organic filler, and silanated colloidal silica (the particle size of inorganic fillers ranges from 0.37 μm to 1.5 μm).	78/40	AB0159
Omnichroma (Tokuyama Dental, Tokyo, Japan)	Mono shade	TEG-DMA, UDMA, Mequinol, Dibutyl hydroxyl toluene, and UV absorber.	Spherical silica–zirconia filler (mean particle size: 0.3 μm, particle size range: 0.2 to 0.4 μm) and composite filler.	79/68	093E82
Vittra APS Unique (FGM, Joinville, SC, Brazil)	Mono shade	Mixture of methacrylate monomers, photoinitator composition (APS), co-initiators, stabilizers, and silane.	Boron–aluminum–silicate glass.	72–80/52–60	040723

Abbreviations: Bis-GMA, 2,2-bis [4-(2-hydroxy-3-methacryloyloxypropoxy) phenyl] propane; TEG-DMA, triethylene glycol di methacrylate; UDMA, urethane dimethacrylate.

**Table 2:** Mean ∆E_00_ values and standard deviations (SD) of composite resins with and without additional light curing.

		800 mW/cm^2^—20 s (QTH)	1000 mW/cm^2^—20 s (LED)	1400 mW/cm^2^—20 s (LED)
Clearfil Majesty ES-2	Single light	2.36 ± 0.16 ^Aa^	2.18 ± 0.2 ^Aa^	1.97 ± 0.44 ^Aa^
	Additional light	2.07 ± 0.3 ^Aa^	1.96 ± 0.44 ^Aa^	1.72 ± 0.21 ^Aa^
Omnichroma	Single light	8.15 ± 1.19 ^Ab^	7.78 ± 1.04 ^ABc^	7.21 ± 0.62 ^Bb^
	Additional light	7.87 ± 1.48 ^Ab^	7.41 ± 1.01 ^Ac^	7.12 ± 0.58 ^Ab^
Vittra APS Unique	Single light	8.3 ± 1.09 ^Ab^	8.28 ± 0.91 ^Ac^	8.11 ± 1.16 ^Ac^
	Additional light	8.11 ± 0.6 ^Bb^	7.84± 0.4 ^Bc^	6.92 ± 1.58 ^Ab^

Abbreviations: LED, light-emitting diode; QTH, Quartz tungsten halogen. Different uppercase superscript letters in rows show a significant difference. Different lowercase superscript letters in the columns show a significant difference. (*p* < 0.05).

**Table 3:** Mean ΔWI_D_ values and standard deviations (SD) of composite resins with and without additional light curing.

		800 mW/cm^2^—20 s (QTH)	1000 mW/cm^2^—20 s (LED)	1400 mW/cm^2^—20 s (LED)
Clearfil Majesty ES-2	Single light	−4.63 ± 0.62 ^Aa^	−3.18 ± 0.39 ^Aa^	−3.12 ± 0.46 ^Aa^
	Additional light	−4.2 ± 0.86 ^Ba^	−2.27 ± 0.62 ^Aa^	−2.21 ± 0.51 ^Aa^
Omnichroma	Single light	−17.14 ± 2.91 ^Ab^	−16.29 ± 2.37 ^Ab^	−14.93 ± 1.68 ^Ac^
	Additional light	−16.65 ± 3.4 ^Bb^	−14.79 ± 2.58 ^Ab^	−14.63 ± 1.36 ^Ac^
Vittra APS Unique	Single light	−16.51 ± 1.08 ^Ab^	−15.62 ± 1.92 ^Ab^	−15.61 ± 1.82 ^Ac^
	Additional light	−15.84 ± 1.88 ^Bb^	−14.81 ± 0.73 ^ABb^	−13.17 ± 3.23 ^Ab^

Abbreviations: LED, light-emitting diode; QTH, Quartz tungsten halogen. Different uppercase superscript letters in rows show a significant difference. Different lowercase superscript letters in the columns show a significant difference. (*p* < 0.05).

**Table 4:** Mean DoC (%) values and standard deviations (SD) of composite resins with and without additional light curing.

		800 mW/cm^2^—20 s (QTH)	1000 mW/cm^2^—20 s (LED)	1400 mW/cm^2^—20 s (LED)
Clearfil Majesty ES-2	Single light	73.26 ± 15.95 ^Aab^	77.80 ± 1.38 ^Aa^	78.19 ± 10.9 ^Aab^
	Additional light	77.97 ± 4.20 ^Aa^	79.32 ± 2.54 ^Aa^	82.58 ± 3.42 ^Aa^
Omnichroma	Single light	58.32 ± 4.88 ^Ac^	65.76 ± 7.37 ^Ab^	74.53 ± 4.52 ^Aab^
	Additional light	65.59 ± 14.24 ^Ab^	66.83 ± 14.42 ^Ab^	78.42 ± 1.64 ^Aab^
Vittra APS Unique	Single light	55.47 ± 1.31 ^Ac^	65.35 ± 11.67 ^Ab^	67.19 ± 3.32 ^Ab^
	Additional light	58.77 ± 9.69 ^Ac^	67.51 ± 7.76 ^Ab^	74.87 ± 10.36 ^Aab^

Abbreviations: LED, light-emitting diode; QTH, Quartz tungsten halogen. Different uppercase superscript letters in rows show a significant difference. Different lowercase superscript letters in the columns show a significant difference. (*p* < 0.05).

## Data Availability

The raw data supporting the conclusions of this article will be made available by the authors on request.
